# Reduced Liver-Specific PGC1a Increases Susceptibility for Short-Term Diet-Induced Weight Gain in Male Mice

**DOI:** 10.3390/nu13082596

**Published:** 2021-07-28

**Authors:** E. Matthew Morris, Roberto D. Noland, Michael E. Ponte, Michelle L. Montonye, Julie A. Christianson, John A. Stanford, John M. Miles, Matthew R. Hayes, John P. Thyfault

**Affiliations:** 1 Department of Molecular & Integrative Physiology, University of Kansas Medical Center, Kansas City, KS 66160, USA; r.noland@rutgers.edu (R.D.N.); mponte@kumc.edu (M.E.P.); jstanford@kumc.edu (J.A.S.); jthyfault@kumc.edu (J.P.T.); 2 Center for Children’s Healthy Lifestyle and Nutrition, Children’s Mercy Hospital, Kansas City, MO 64108, USA; 3 Department of Nutrition & Exercise Physiology, University of Missouri, Columbia, MO 65211, USA; michelle.gastecki@gmail.com; 4 Department of Anatomy and Cell Biology, University of Kansas Medical Center, Kansas City, KS 66160, USA; jchristianson@kumc.edu; 5 Department of Internal Medicine—Division of Endocrinology and Metabolism, University of Kansas Medical Center, Kansas City, KS 66160, USA; jmiles3@kumc.edu; 6 Department of Psychiatry, University of Pennsylvania, Philadelphia, PA 19104, USA; hayesmr@pennmedicine.upenn.edu; 7 Kansas City VA Medical Center-Research Service, Kansas City, MO 64128, USA

**Keywords:** energy homeostasis, weight gain, food intake, energy expenditure, liver, mitochondria

## Abstract

The central integration of peripheral neural signals is one mechanism by which systemic energy homeostasis is regulated. Previously, increased acute food intake following the chemical reduction of hepatic fatty acid oxidation and ATP levels was prevented by common hepatic branch vagotomy (HBV). However, possible offsite actions of the chemical compounds confound the precise role of liver energy metabolism. Herein, we used a hepatocyte PGC1a heterozygous (LPGC1a) mouse model, with associated reductions in mitochondrial fatty acid oxidation and respiratory capacity, to assess the role of liver energy metabolism in systemic energy homeostasis. LPGC1a male, but not female, mice had a 70% greater high-fat/high-sucrose (HFHS) diet-induced weight gain compared to wildtype (WT) mice (*p* < 0.05). The greater weight gain was associated with altered feeding behavior and lower activity energy expenditure during the HFHS diet in LPGC1a males. WT and LPGC1a mice underwent sham surgery or HBV to assess whether vagal signaling was involved in the HFHS-induced weight gain of male LPGC1a mice. HBV increased HFHS-induced weight gain (85%, *p* < 0.05) in male WT mice, but not LPGC1a mice. These data demonstrate a sex-specific role of reduced liver energy metabolism in acute diet-induced weight gain, and the need for a more nuanced assessment of the role of vagal signaling in short-term diet-induced weight gain.

## 1. Introduction

Understanding the homeostatic mechanisms that mediate susceptibility to weight gain is becoming more critical as the prevalence of obesity continues to rise [1]. Indeed, the prevention of weight gain may be a more successful strategy to combat the obesity epidemic in light of the evidence that losing and maintaining body weight is difficult [2]. Considering the etiology of being overweight and obesity, it is worth noting that long-term weight gain commonly results in the culmination of small, sporadic bouts of weight gain occurring over long time frames [3,4,5,6] as a function of metabolic, hedonic, hormonal, and satiety driven mechanisms [7,8,9,10]. Among these mechanisms is a growing list of peripheral hormonal and afferent neural signals that are integrated in the brain and impact whole-body energy homeostasis [11].

The regulation of energy balance is complex and multifactorial; energy intake and energy expenditure are independently and dependently regulated [12]. Both energy intake and expenditure are modulated by the central action of peripheral hormones (e.g., leptin, ghrelin, and insulin) and the integration of peripheral neural signals. Previous findings showed that the inhibition of hepatic fatty acid oxidation with intraperitoneally injected chemical inhibitors acutely increased food intake [13,14,15]. Further, these findings suggested that liver energy metabolism could influence energy intake via afferent vagal signals [13,14,16,17], as the hyperphagic effects produced by reduced hepatic fatty acid oxidation could be eliminated if vagal communication was disrupted by common hepatic branch vagotomy [15,16]. However, these findings were potentially confounded by the off-target effects of the chemical inhibitors.

To more specifically target liver energy metabolism with molecular techniques, we utilized a mouse model with a liver-specific reduction in the *pgc1a* gene (LPGC1a) [18]. PGC1a (peroxisome proliferator-activated receptor g-1a) is a key regulator of mitochondrial biogenesis and fatty acid oxidation in the liver [19] and serves as a transcriptional node for energy/nutrient signaling pathways such as fasting and reduced energy status (ATP/ADP ratio) [20,21]. We have previously observed that male LPGC1a mice have reduced liver homogenate fatty acid oxidation and similar energy expenditure and food intake during chow feeding compared to WT mice [22]. Others have observed that the LPGC1a mouse has a reduced expression of hepatic fatty acid oxidation genes, which was associated with increased steatosis and circulating triglycerides [18]. This is opposite to the effect we have previously reported with hepatic PGC1a overexpression, which increased fat oxidation and mitochondrial capacity [19]. Using the LPGC1a mouse, we tested the hypothesis that a reduced liver mitochondrial fatty acid oxidation and respiratory capacity would result in greater weight gain during exposure to short-term high-fat, high-sucrose feeding in male and female mice. Our findings support an association between decreased liver energy metabolism and the dysregulation of energy homeostasis during short-term high-fat, high-sucrose feeding in male, but not female, mice. Moreover, additional common hepatic branch vagotomy experiments support the role of the vagal nerve in energy homeostasis, but do not clarify whether the communication of liver energy metabolism to the brain is a necessary component.

## 2. Materials and Methods

### 2.1. Animals

The animal protocol was approved by the Institutional Animal Care and Use Committee at the University of Missouri, Harry S Truman Memorial Veterans’ Hospital (ACUC #7758), and the University of Kansas Medical Center (ACUP 2019-2484). All experiments were carried out in accordance with the *Guide for the Care and Use of Laboratory Animals* published by the US National Institutes of Health (NIH guide, 8th edn, 2011). Mice were anaesthetized with pentobarbital sodium (75 mg/kg) before a terminal procedure. Mice with a predominant liver PGC1a heterozygosity (LPGC1a) were produced as previously described [18,22,23]. Briefly, C57Bl/6J male mice (#000664, Jackson Laboratory, Bar Harbor, ME, USA) were bred to female homozygous *pgc1a* floxed mice (#00966, B6N.129(FVB)-*Ppargc1a^tm2.1Brsp^*/J, Jackson Laboratory, Bar Harbor, ME, USA) to produce heterozygous *pgc1a* floxed offspring. Female heterozygous *pgc1a* floxed mice were subsequently bred to male mice with the transgenic expression of the *cre* recombinase gene under control of the albumin promoter (#003574, B6.Cg-*Speer6-ps1^TG(Alb-cre)21Mgn^*/J, Jackson Laboratory, Bar Harbor, ME, USA). The resultant littermates all expressed the albumin-cre transgene, and were either homozygous (wildtype (WT), +/+) or heterozygous for *pgc1a* in a liver-specific manner (LPGC1a, +/−). Mice were housed at ~25–27 °C on a 12/12 light/dark cycle, with ad lib access to water and a low-fat diet (LFD; D12110704: 10% kcal fat, 70% kcal carbohydrate (3.5% kcal sucrose), 20% kcal protein, and 3.85 kcal/g energy density, Research Diets, New Brunswick, NJ, USA). All mice were fasted for 4 h prior to euthanasia and tissue collection. Livers were quickly removed following exsanguination, and either stored in an ice-cold homogenization buffer (100 mM KCl, 40 mM Tris·HCl, 10 mM Tris-base, 5 mM MgCl_2_·6H_2_O, 1 mM EDTA, and 1 mM ATP; pH 7.4) or were snap frozen in liquid N_2_ for storage at −80°C.

### 2.2. Genotyping and RT-PCR

**Figure 1:**
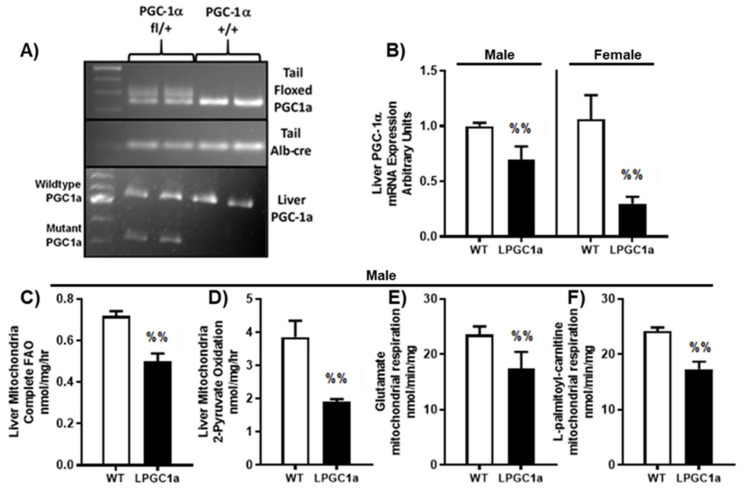
Liver-specific PGC1a heterozygosity results in reduced fatty acid oxidation and oxidative capacity in isolated liver mitochondria. (**A**) Tail and liver genotyping of WT and LPCG1a mice. (**B**) Relative liver mRNA expression of PGC1a. Complete oxidation of 1-[14C]-palmitate (**C**) and 2-[14C]-pyruvate (**D**) to CO_2_ in isolated liver mitochondria in male mice. Isolated liver mitochondrial respiratory capacity was determined in male mice by the measurement of O_2_ consumption using a Clark electrode system in the presence of (**E**) glutamate (+malate) and (**F**) L-palmitoyl-carnitine (+malate) during state 3 respiration (+ADP). Values are means ± SEM (*n* = 4–6). %% *p* < 0.05 WT vs. LPGC1a for *t*-tests.

Mouse and liver-specific genotypes were confirmed as previously described [18,24]. A list of appropriate genotyping primers is provided in Table 1, and representative images of the genotyping are presented in Figure 1A. RNA was isolated from ~20–30 mg of liver tissue using the RNeasy Plus Mini Kit (Qiagen, Valencia, CA, USA), with cDNA produced using the ImProm-II RT system (Promega, Madison, WI, USA). A real-time quantitative PCR was performed using a Prism 7000 (Applied Biosystems, Foster City, CA, USA) and SYBR Green. The SYBR Green primers for PGC1a are provided in Table 1. All gene specific values were normalized to Cyclophilin B (*ppib*) expression.

### 2.3. Liver Isolated Mitochondria Fatty Acid and Pyruvate Oxidation

Liver mitochondrial fatty acid oxidation and the pyruvate oxidation was performed in isolated liver mitochondria as previously described [19]. To isolate the liver mitochondria, approximately 1 g of liver was crude minced in 8 mL of an ice-cold mitochondrial isolation buffer (220 mM mannitol, 70 mM sucrose, 10 mM Tris, 1 mM EDTA, pH adjusted to 7.4 with KOH). Minced tissue was homogenized with a 15 mL glass-on-Teflon homogenizer (8 passes at 2000 rpm). The crude homogenate was created by centrifugation (4 °C, 10 min, 1500× *g*). The supernatant was transferred to a round bottom tube and centrifuged (4 °C, 10 min, 8000× *g*). The pellet was resuspended in 6 mL of the isolation buffer using a Dounce glass-on-glass homogenizer and centrifuged again (4 °C, 10 min, 6000× *g*). The pellet was resuspended in 4 mL of the isolation buffer containing 0.1% BSA and centrifuged (4 °C, 10 min, 4000× *g*). This final pellet was resuspended in ~0.75 mL of the isolation buffer. The protein concentration for both suspensions was determined by BCA assay. The complete oxidation of [1-14C]-palmitate and [2-14C]-pyruvate to CO_2_ was measured in the liver mitochondria (*n* = 4, male), as previously described [19]. Briefly, the fatty acid and pyruvate oxidation was assessed by the measurement of the production of ^14^CO_2_ in a sealed trapping device containing 20 mM palmitate ([1-14C]-palmitate) or 5 mM pyruvate ([2-14C]-pyruvate), a tissue sample, and a reaction buffer (100 mM sucrose, 10 mM Tris·HCl, 10 mM KPO_4_, 100 mM KCl, 1 mM 4 MgCl_2_·6H_2_O, 1 mM L-carnitine, 0.1 mM malate, 2 mM ATP, 0.05 mM CoA, and 1 mM DTT, pH 7.4) at 37 °C.

### 2.4. Mitochondrial Respiration

The mitochondrial respiration of the substrates glutamate and palmitoyl-carnitine was measured in isolated liver mitochondria (*n* = 4, male), as previously described [25]. Briefly, liver mitochondria oxygen consumption was assessed using a Clark-type electrode system (Strathkelvin Instruments, North Lanarkshire, Scotland). Incubations were carried out at 37 °C in a 0.5 mL final volume containing 100 mM KCl, 50 mM MOPS, 10 mM K_2_PO_4_, 10 mM MgCl_2_, 0.5 mM EGTA, 20 mM glucose, and a 0.2% bovine serum albumin with a pH of 7.4. The complex I-mediated mitochondrial respiration of either the 10 mM glutamate or the 10 mM L-palmitoyl-carnitine was monitored in the presence of 1 mM malate and 100 mM ADP. Oxygen consumption (in nmol/min) was normalized to the mitochondrial protein in the respirometer cell.

### 2.5. Indirect Calorimetry Experiments

Energy metabolism was assessed in male and female LPGC1a and WT littermate mice (14–16 weeks of age, n = 7–10) for 3 days on either the LFD or high-fat/high-sucrose diet (HFHS, D12451: 45% kcal fat, 35% kcal carbohydrate (17% kcal sucrose), 20% kcal protein, and 4.73 kcal/g energy density, Research Diets, New Brunswick, NJ, USA) by measuring VO2 in a Promethion continuous metabolic monitoring system (Sable Systems International, Las Vegas, NV, USA), as previously described [22]. Animals were acclimated to the indirect calorimetry cages for at least 4 days prior to the start of the dietary intervention and data collection. Animal body weight and food weight were assessed prior to and following the 3 day indirect calorimetry data collection. The rate of energy expenditure (EE) was calculated as EE (kcal/h) = 4.934 X VO2 [26], with the total EE calculated as the rate of EE times 24 (per day) and summed across the 3 days of analysis. Resting EE was calculated as the average rate of EE (kcal/h) during the daily 30 min period with the lowest EE times 24 (per day) and summed across the intervention. Non-resting EE is the difference in total and resting EE. Energy intake was calculated across the 3 day intervention as food intake (grams) times the energy density of the two diets (kcal/g). Energy balance was determined as the difference in energy intake and total EE. As previously described [27], the thermic effect of food was determined as the consensus thermic effect of each macronutrient times the manufacturer provided diet information. From this, the thermic effect of food for the LFD (D12110704, Research Diets, 3.85 kcal/g, 10% kcals fat, 65% kcal carbohydrate, 20% kcals protein) was 10.5% or 0.4043 kcal/g, and the HFHS diet (D12451, Research Diets, 4.73 kcal/g, 45% kcals fat, 35% kcal carbohydrate, 20% kcals protein) was 8.75% or 0.4139 kcal/g. Activity EE is the difference between the non-resting EE and the thermic effect of food. All_meters is an assessment of cage activity and is calculated using the summed distances determined from mouse movements based on XY beam breaks. The cost of movement is a calculation of the energy efficiency of movement and is determined by the activity EE divided by All_meters.

### 2.6. Common Hepatic Branch Vagotomy (HBV) Experiments

#### 2.6.1. Surgery

Starting at 10–12 weeks of age, male (*n* = 7–12) and female (*n* = 6–9) LPGC1a and WT littermates underwent common hepatic branch vagotomy or sham surgery under isoflurane as previously reported [28]. Briefly, a ventral midline incision was made in the abdominal skin and muscle. The ligaments holding the liver lobes were dissected allowing the liver to be everted towards the diaphragm, clearly exposing the esophagus and stomach. The stomach was gently retracted and the common hepatic branch of the vagus was identified as it branches from the descending vagus nerve along the esophagus and runs near the hepatoesophageal artery. For mice in the vagotomy group, the HBV was ablated by cauterization. The abdominal wall and skin were subsequently sutured with 5-0 or 6-0 Vycril. Mice received a single dose of buprenorphine (1.0 mg/kg SQ) for analgesia. Mice were individually housed and daily post-operative monitoring included food intake and body weight assessment for 7 days. Mice had ad lib access to an LFD throughout the initial recovery observations and until the HFHS weight gain studies began 2–3 weeks later.

#### 2.6.2. HFHS-Induced Weight Gain and Body Composition

The one-week HFHS (D12451) feeding studies began 3–4 weeks post-surgery. The short-term diet intervention was lengthened from 3 days to one week as an attempt to decrease the variability in outcomes. Body weights, food weights, and body composition were assessed prior to and post 7 days of exposure to the HFHS diet. Body composition was determined by qMRI (EchoMRI-1100, EchoMRI, Houston, TX, USA), with the fat-free mass calculated as the difference between body weight and fat mass. As above, energy intake was determined from the food intake and energy density of the diet. Feed efficiency was calculated as the change in body weight divided by the energy intake. The metabolic efficiency percentage was calculated as previously described and is determined as the sum of the change in fat mass (kcal) and the change in fat-free mass (kcal) divided by the energy intake [29].

### 2.7. Statistical Analysis

Data are presented as means and standard error. The two-standard deviation test was used to test for outliers in each group and outliers were excluded from the data analysis. A two-way ANOVA was used to determine the interactions and the main effects of genotype and diet or genotype and surgery on data separately for each sex. Where significant interactions or main effects were observed, a post hoc analysis was performed using Fisher’s least significant difference to test for any specific pairwise differences using SPSS version 25 (SPSS Inc., Armonk, New York, NY, USA). The main effects are discussed only when all pairwise treatment comparisons within that parameter were significant. In three instances, Figures 4D, 5A and 7F, a one-way ANOVA with least significant differences post-hoc was used to assess the differences between groups that lacked a significant two-way interaction or a main effect. For Figure 1C–F, a one-tailed t-test was used to compare the WT and LPGC1a values. ANCOVA was used to adjust energy intake data for differences in initial body weight observed in male mice of HBV experiments. Estimated marginal means and effect size (partial eta squared) are reported.The statistical significance was set at *p* < 0.05.

## 3. Results

### 3.1. Liver-Specific PGC1a Heterozygous Mice Have Reduced Mitochondrial Fatty Acid Oxidation and Respiratory Capacity

As previously observed [18], the breeding of female heterozygous floxed pgc1a mice with male albumin-cre recombinase mice produced liver-specific, pgc1a heterozygous and wild-type (WT) littermate mice (Figure 1A). Male and female liver PGC1a mRNA expression was ~30% and 70% lower in LPGC1a mice compared to WT mice, respectively (Figure 1B, *p* < 0.05). The initial experiments in male mice demonstrate that this reduced liver PGC1a expression led to a ~30% less complete fatty acid oxidation to CO_2_ and a ~50% less oxidation of pyruvate to CO_2_ in isolated liver mitochondria (Figure 1C,D, respectively, *p* < 0.05). Additionally, male LPGC1a mice displayed a ~30% reduction in the ADP-dependent respiration of glutamate/malate and palmitoyl-carnitine/malate in liver mitochondria compared to WT mice (Figure 1E,F, respectively, *p* < 0.05).

### 3.2. Reduced Liver PGC1a Increases Susceptibility to Short-Term HFHS Weight Gain in Male, but Not Female, Mice

**Figure 2:**
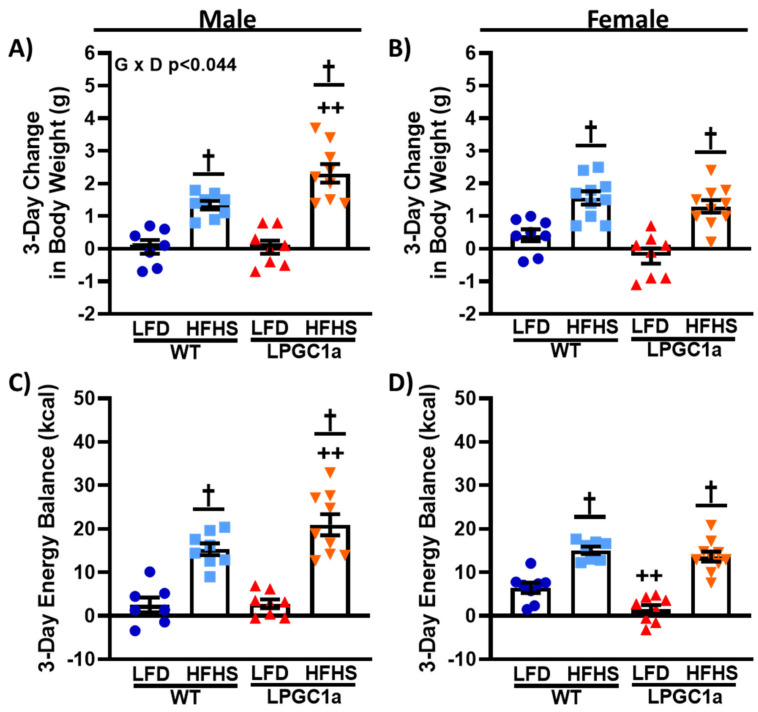
Male LPGC1a mice have greater short-term HFHS-induced weight gain. Body weight gain was assessed during the 3 days of the LFD or the HFHS diet in male (**A**) and female (**B**) WT and LPGC1a mice. Energy balance during the 3 day feeding intervention was determined as energy intake minus total EE for male (**C**) and female (**D**) mice. Individual data points and the group means ± SEM are displayed (*n* = 7–10). Genotype Diet *p* < 0.05 interaction of genotype and diet, † *p* < 0.05 main effect of diet, ++ *p* < 0.05 WT vs. LPGC1a within diet.

To assess whether liver-specific reductions in PGC1a altered energy homeostasis during short-term HFHS diet exposure, weight gain, and energy balance were assessed in male and female LPGC1a and WT littermates following a 3 day HFHS dietary challenge. While females weighed less than males, no difference by genotype was observed in the initial body weight in either sex before the 3 day HFHS diet (Table 2). On the LFD, there was no difference in 3 day weight gain between male WT and LPGC1a mice (Figure 2A). As expected, there was a significant main effect for HFHS-induced weight gain in male mice compared to the LFD (*p* < 0.05). Importantly, the male LPGC1a mice had ~>70% greater weight gain during the 3 days of HFHS exposure compared to WT mice (*p* < 0.05), resulting in a significant interaction of diet and genotype (*p* < 0.035) (Figure 2A). Interestingly, while female WT mice gained ~1.5-fold more weight during the 3 days of the LFD compared to LPGC1a mice (*p* < 0.05, Figure 2B), HFHS feeding resulted in the same weight gain in female mice regardless of genotype (*p* < 0.05). The energy balance was not different in male mice fed LFD (Figure 2C), with HFHS feeding resulting in a 6- and 8-fold more positive energy balance in WT and LPGC1a male mice, respectively (*p* < 0.05). LPGC1a male mice had a ~35% more positive energy balance during HFHS feeding than WT mice (*p* < 0.05). As with weight gain, female LPGC1a mice had a lower energy balance on the LFD compared to WT mice (~80%, Figure 2D, *p* < 0.05), and short-term HFHS exposure resulted in a more positive energy balance in both female WT and LPGC1a mice (2.5- and 9-fold, respectively, *p* < 0.05). These data demonstrate that increased susceptibility to acute weight gain in male mice with liver-specific reductions in PGC1a is due to exaggerated HFHS-induced impairments in energy homeostasis regulation.

### 3.3. Male LPGC1a Mice Have Slight Increases in HFHS Intake and Altered Feeding Behaviors

**Figure 3:**
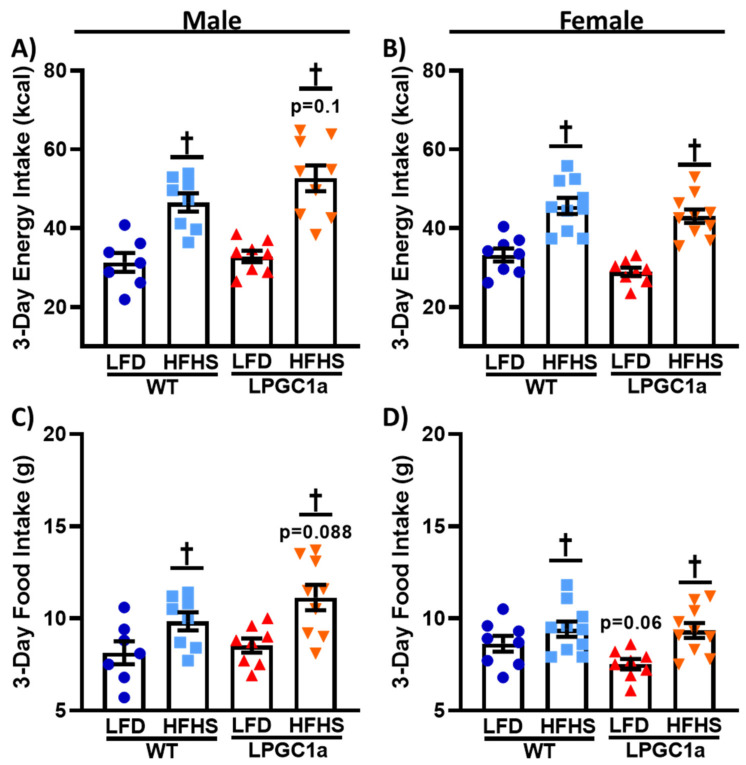
Reduced liver PGC1a subtly impacts HFHS food and energy intake in male mice. (**A**,**B**) Energy intake during the 3 days of indirect calorimetry was determined as the energy density of each diet (kcal/g) times the total (**C**,**D**) food intake (g) for male and female WT and LPGC1a mice, respectively. Individual data points and the group means ± SEM are displayed (*n* = 7–10). † *p* < 0.05 main effect of diet.

To determine the factors resulting in the greater weight gain and positive energy balance in male LPGC1a mice on the HFHS diet, an analysis of energy intake was performed. The energy intake was not different for LFD fed male mice, regardless of genotype (Figure 3A). A total of 3 days of HFHS feeding resulted in a ~50% and ~60% greater energy intake in male WT and LPGC1a mice, respectively (*p* < 0.05), with LPGC1a males on a HFHS diet tending to have a greater energy intake compared to WT mice (~13%, *p* = 0.09). Female LPGC1a mice tended to have a lower energy intake compared to WT mice (~6%, Figure 3B, *p* = 0.1), and exposure to a HFHS diet resulted in a ~40% and ~50% increase in energy intake in female WT and LPGC1a mice, respectively, compared to the mice that were fed an LFD (*p* < 0.05). To assess whether any observed differences in energy intake were due to differences in the absolute consumption of the two diets, food intake during the 3 day intervention was analyzed. In Figure 3C, male WT and LPGC1a mice consumed the same amount of the LFD, while consuming more of the HFHS diet during the short-term exposure (~20% and ~30%, respectively, *p* < 0.05). As expected, based on energy intake data, male LPGC1a mice tended to have greater intake of the HFHS diet compared to WT mice (~13%, *p* = 0.1). Female WT and LPGC1a mice also had an increased food intake on the HFHS diet (~12% and ~20%, respectively, Figure 3D, *p* < 0.05), with LFD LPGC1a mice tended to have a reduced intake compared to WT mice (~10%, *p* = 0.06). These data suggest that the greater weight gain induced by the HFHS diet in the male LPGC1a mice is partially due to differences in energy intake regulation.

**Figure 4:**
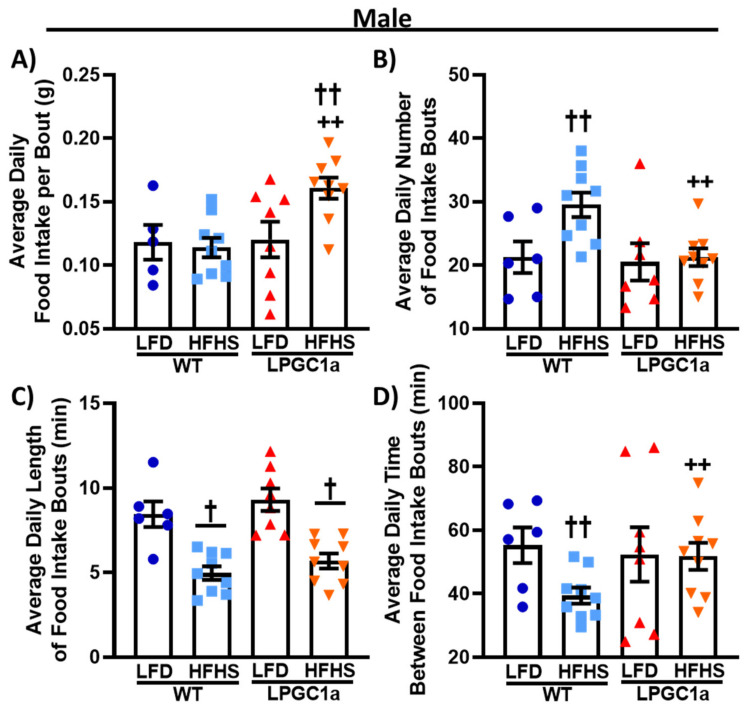
Male LPGC1a mice altered feeding patterns during short-term HFHS feeding. Feeding patterns during the 3 day indirect calorimetry experiments were evaluated in male WT and LPCG1a mice on an LFD and a HFHS diet for (**A**) food intake per feeding bout (g), (**B**) number of daily feeding bouts, (**C**) length of feeding bouts (min), and (**D**) time interval between successive feeding bouts (min). Individual data points and the group means ± SEM are displayed (*n* = 7–10). † *p* < 0.05 main effect of diet, †† *p* < 0.05 LFD vs. HFHS within genotype, ++ *p* < 0.05 WT vs. LPGC1a within diet.

To specifically investigate the subtle increases observed in food and energy intake only observed between HFHS-fed male LPGC1a and WT mice, we assessed their feeding behaviors during the indirect calorimetry experiments (Figure 4). Male HFHS-fed LPGC1a mice consumed ~35% more food per feeding bout than those on the LFD (Figure 4A, *p* < 0.05), and ~40% more than HFHS-fed WT mice (*p* < 0.05). No difference in grams of food intake per feeding bout were observed between mice fed on the LFD. Again, no difference was observed between mice fed on the LFD when assessing the average number of daily feeding bouts (Figure 4B). However, the HFHS-fed WT male mice had a ~40% increase in the number of daily feeding bouts compared to the LFD (*p* < 0.05), and a ~30% increase compared to HFHS-fed LPGC1a mice (*p* < 0.05). No difference was observed for the number of feeding bouts between LPGC1a mice on the LFD and the HFHS diet. The length of the feeding bouts was not different between LFD-fed male WT and LPGC1a mice; however, HFHS feeding resulted in a ~40% reduction in the length of feeding bouts for both WT and LPGC1a mice (Figure 4C, *p* < 0.05). Finally, the average time between feeding bouts was reduced by ~30% in HFHS-fed male WT mice compared to WT LFD and LPGC1a HFHS (Figure 4D, *p* < 0.05). No difference in the length of time between the feeding bouts was observed between the LPGC1a male mice on the LFD and the HFHS diet. Further, no differences were observed in feeding behavior between female WT and LPGC1a mice (data not shown). Overall, these data demonstrate that a reduced liver PGC1a alters feeding behaviors in male mice during short-term exposure to a HFHS diet.

### 3.4. Lower Non-Resting EE in HFHS-Fed LPGC1a Male Mice Prevents HFHS-Induced Total EE Increase

**Figure 5:**
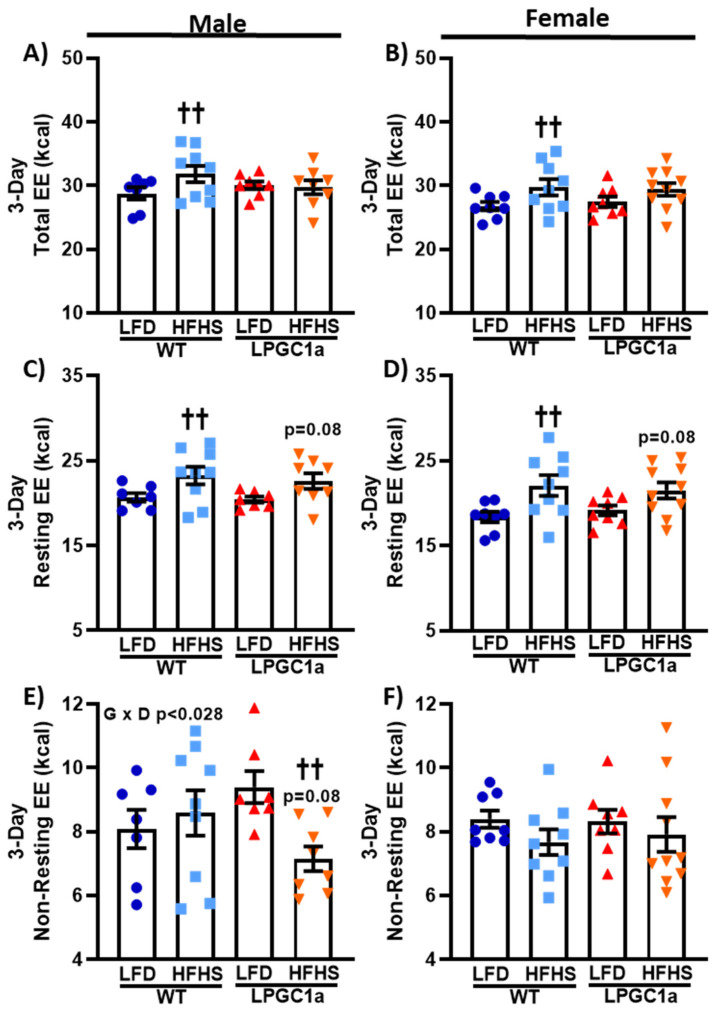
Reduced liver PGC1a blunt HFHS-induced increases in total EE in male mice due to lower non-resting EE. Indirect calorimetry during the 3 day diet intervention was used to determine total EE and its primary components: resting EE and non-resting EE in male and female WT and LPCG1a mice. Male and female data are presented for total EE (**A**,**B**), resting EE (**C**,**D**), and non-resting EE (**E**,**F**), respectively. Individual data points and the group means ± SEM are displayed (*n* = 7–10). Genotype x Diet *p* < 0.05 interaction of genotype and diet, †† *p* < 0.05 LFD vs. HFHS within genotype.

The observed differences in male weight gain in the LPGC1a mice could also be impacted by differences in EE. Therefore, we analyzed the total EE and its primary components, resting- and non-resting EE, in male and female WT and LPGC1a mice (Figure 5). The total EE was ~10% greater in male WT HFHS-fed mice compared to mice that were fed the LFD (Figure 5A, *p* < 0.05), an effect that did not occur in the LPGC1a mice. The total EE was not different between LFD-fed WT mice and LPGC1a mice regardless of sex (Figure 5A,B). Similarly, female WT mice had a ~10% greater total EE when fed the HFHS diet compared to the LFD (Figure 5B, *p* < 0.05), which was not different from the total EE of HFHS-fed LPGC1a female mice. Moreover, the EE was not different between female LPGC1a mice on the LFD and the HFHS diet. As the largest component of total EE, the resting EE represents the EE of the basal metabolic rate and any adaptive thermogenesis. The resting EE was ~10% greater in male WT mice on the HFHS diet compared to the LFD (Figure 5C, *p* < 0.05). Male HFHS-fed LPGC1a mice tended to have a greater resting EE compared to the mice that were fed the LFD (*p* = 0.08). Likewise, female HFHS-fed WT mice had a ~20% greater resting EE compared to LFD-fed mice (Figure 5D, *p* < 0.05). Again, the resting EE tended to be higher in female HFHS-fed LPGC1a mice compared to mice that were fed the LFD (*p* = 0.08. Non-resting EE represents 20–50% of the total EE and is comprised primarily of the thermic effect of food and the EE of activity. No difference in the non-resting EE was observed between WT males fed the LFD and the HFHS diet, or between LFD-fed males of both genotypes. Interestingly, HFHS feeding reduced the non-resting EE in male LPGC1a mice by ~25% compared to the LFD (Figure 5E, *p* < 0.05), and tended be lower compared to HFHS-fed WT males (~17%, *p* = 0.08). Together, these differences produced a significant interaction (*p* < 0.028) of genotype and diet in male mouse non-resting EE. No difference in the non-resting EE was observed in female mice in either genotype or diet (Figure 5F). These data demonstrate that male LPGC1a mice have a mal-adaptive response in non-resting EE upon short-term exposure to a HFHS diet, which limits or eliminates the diet-induced adaptive response of the total EE.

### 3.5. Male LPGC1a Mice Fed a HFHS Diet Have Reduced Activity EE and Home Cage Activity

**Figure 6:**
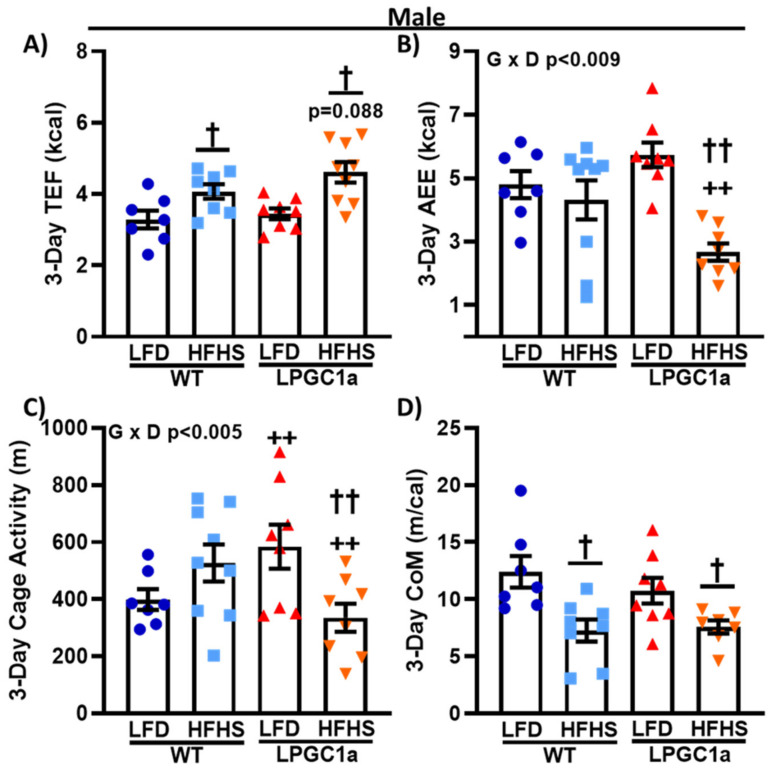
Male LPGC1a mice have lower cage activity and activity EE during short-term HFHS feeding. (**A**) Thermic effect of food was calculated based on the thermic effect of each macronutrient in each diet multiplied by the amount of food intake. (**B**) Activity EE was calculated as the non-resting EE minus the thermic effect of food. Cage activity is represented as total meters traveled (All_Meters, (**C**)). The energy efficiency of movement or cost of movement (**D**) was calculated as the activity EE divided by the cage activity. Individual data points and the group means ± SEM are displayed (*n* = 7–10). Genotype x Diet *p* < 0.05 interaction of genotype and diet, † *p* < 0.05 main effect of diet, †† *p* < 0.05 LFD vs. HFHS within genotype, ++ *p* < 0.05 WT vs. LPGC1a within diet.

To focus on the diet-induced difference in non-resting EE in male LPGC1a mice, we also assessed the components of non-resting EE: thermic effect of food and activity EE. The thermic effect of food data mimics food/energy intake (Figure 3). No differences were observed between LFD-fed male mice (Figure 6A) and a ~25% and ~35% increase was observed in HFHS-fed male WT and LPGC1a mice, respectively (*p* < 0.05). Moreover, as with food/energy intake, male HFHS LPGC1a mice tended to have a greater thermic effect of food compared to WT mice (*p* = 0.1). Male HFHS-fed LPGC1a mice had a ~40% lower activity EE compared to WT mice and ~50% compared to the mice that were fed the LFD, which resulted in a significant interaction of genotype and diet (*p* < 0.009) (Figure 6B). Because of the differences in activity EE in male HFHS-fed LPGC1a mice, we also quantified home cage activity. Male LPGC1a mice on an LFD had a ~45% greater cage activity compared to WT mice (*p* < 0.05) (Figure 6C). Importantly, male HFHS-fed LPGC1a mice had a ~35% lower home cage activity compared to WT mice (*p* < 0.05), and a ~40% lower home cage activity compared to LFD-fed LPGC1a mice (*p* < 0.05). We also calculated the energy cost of movement as the activity EE divided by the distance moved during cage activity (Figure 6D). HFHS feeding reduced the cost of movement by ~40% and ~30% in male WT and LPGC1a mice, respectively (*p* < 0.05). Finally, no differences were observed in the non-resting EE components between female WT and LPGC1a mice (data not shown). These data demonstrate that a reduced liver PGC1a in male mice results in reductions in activity level and the associated EE during short-term exposure to a HFHS diet.

### 3.6. Common Hepatic Branch Vagotomy Increases Short-Term HFHS-Induced Weight Gain in WT Mice, but Not in LPGC1a Mice

**Figure 7:**
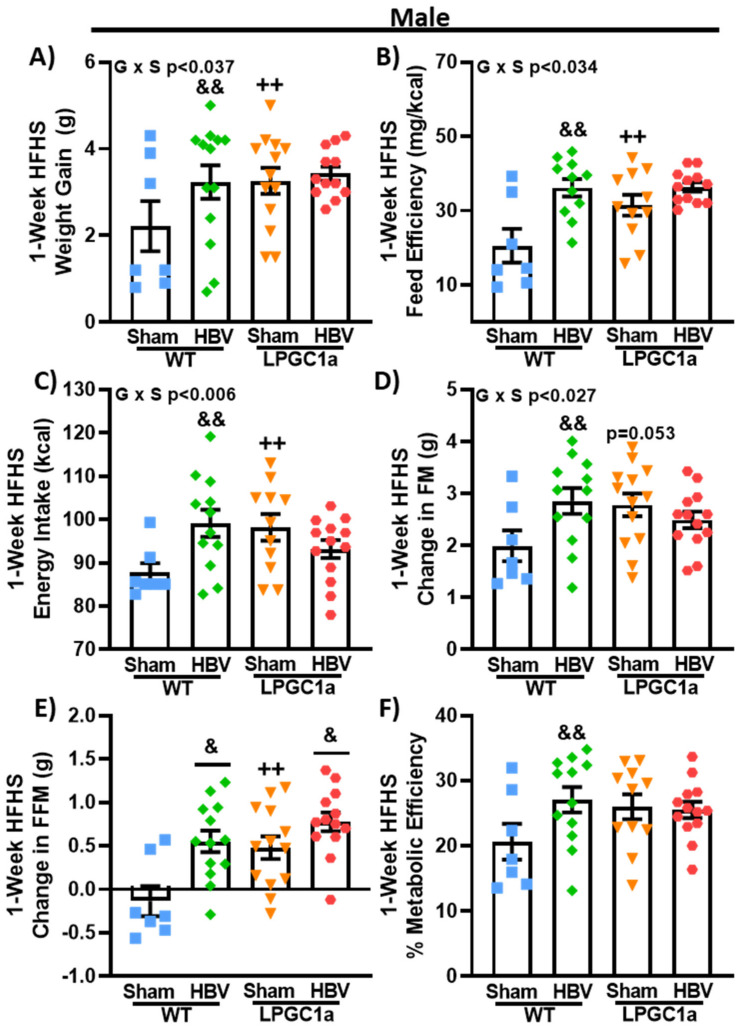
Disruption of liver vagal innervation by common hepatic branch vagotomy increases short-term diet-induced weight gain in WT, but not LPGC1a, male mice. Male WT and LPGC1a mice underwent sham or HBV surgery, and were administered a one-week HFHS diet following recovery. (**A**) One-week HFHS-induced weight gain. (**B**) Feed efficiency was determined as the body weight gained divided by the energy intake during the one-week HFHS diet. (**C**) Energy intake was calculated as the energy density of the HFHS diet multiplied by the one-week food intake. A quantitative MRI was utilized to determine fat- and fat-free mass at the initiation and end of the one-week HFHS diet, and the change in fat mass (**D**) and the fat-free mass (**E**) are presented. Based on the change in fat- and fat-free mass the (**F**) metabolic efficiency of weight gain was calculated as the sum of the energy content of fat and fat-free mass multiplied by the respective changes over the one-week HFHS diet, divided by the one-week energy intake. Individual data points and the group means ± SEM are displayed (*n* = 7–12). Genotype x Surgery *p* < 0.05 interaction of genotype and surgery, & *p* < 0.05 main effect of HBV, && *p* < 0.05 sham vs. HBV within genotype, ++ *p* < 0.05 WT vs. LPGC1a within surgery.

Previous work has described a hepatic vagal afferent neural pathway that is involved in the regulation of acute food intake [15,30] and insulin secretion [31,32]. We performed a common hepatic branch vagotomy (HBV) or sham surgery to test if the hepatic vagal afferent pathway was mediating the increased short-term diet-induced weight gain observed in male LPGC1a mice. Following at least two weeks of recovery from the surgical procedure, we assessed body weight gain, energy intake, and changes in body composition for one week (7 days) on an LFD followed by 1 week on the HFHS diet in both WT and LPGC1a male mice. No differences were observed in body weight, energy intake, or body composition between the genotypes or the mice that underwent surgery at the initiation of experiments (data not shown) or during the LFD (data not shown). At the initiation of the one-week HFHS diet, sham LPGC1a males tended (Table 2, *p* = 0.06) to weigh more than sham WT mice and were significantly heavier than HBV LPGC1a mice (*p* < 0.05). No difference was observed in the initial body weight between the WT sham mice and HBV mice. As with the earlier experiments, male sham LPGC1a mice gained ~70% more weight during one-week of HFHS feeding compared to sham WT mice (Figure 7A, *p* < 0.05). Male HBV WT mice gained ~85% more weight during HFHS feeding compared to sham WT (*p* < 0.05), while the weight gain in HBV LPGC1a mice was not different from that of the sham LPGC1a mice (Figure 7A). Feed efficiency (weight gained divided by energy intake) tracked with the weight gain data (Figure 7B). Feed efficiency in sham LPGC1a mice was ~50% higher than in sham WT mice (*p* < 0.05). HBV WT mice had a ~75% greater feed efficiency compared to sham WT mice (*p* < 0.05). Energy intake during the one-week HFHS diet was ~12% greater in HBV WT mice and ~9% greater in sham LPGC1a mice compared to sham WT mice (Figure 7C, *p* < 0.05). Due to the differences in initial body weight, ANCOVA was used to calculate the estimated marginal means adjusting for body weight. Body weight was a significant co-variate (*p* < 0.001) but had a relatively low effect size (partial eta squared—0.244). The estimated marginal means (WT sham 87.5 ± 3.0 kcal, WT HBV 100.6 ± 2.3 kcal, LP sham 93.8 ± 2.7 kcal, LP HBV 95.7 ± 2.3 kcal) were similar to the absolute values (Figure 7C). However, only the increased energy intake of WT HBV mice compared to sham WT mice was significant (*p* < 0.05). Body composition was determined in these experiments utilizing qMRI. Short-term HFHS-induced changes in fat mass tracked well with energy intake (Figure 7D), with HBV WT mice gaining ~40% fat mass compared to sham WT mice and sham LPGC1a mice tending to have greater fat mass gain than sham WT mice (~30%, *p* = 0.053). Again, the HBV in LPGC1a mice did not impact fat mass. Interestingly, short-term HFHS-induced changes in fat-free mass were ~7- and ~2-fold greater in HBV WT and HBV LPGC1a mice, respectively (Figure 7E, *p* < 0.05) compared to sham mice. In addition, there was a ~4.5-fold greater increase in FFM in sham LPGC1a mice compared to sham WT mice (*p* < 0.05). Finally, to specifically assess the allocation of stored energy, metabolic efficiency was calculated as the energy content of the gained fat- and fat-free mass divided by energy intake (Figure 7F) [29]. HBV only significantly increased metabolic efficiency in WT mice (~30%, *p* < 0.05), and did not increase metabolic efficiency in LPGC1a mice. These data highlight that the loss of the hepatic vagal afferent neural pathway is sufficient to increase short-term HFHS-induced weight gain in male mice, but it does not resolve the male LPGC1a diet-induced weight gain phenotype.

### 3.7. Common Hepatic Branch Vagotomy Divergently Impacts Short-Term HFHS-Induced Fat Mass Gains in Female WT and LPGC1a Mice

**Figure 8:**
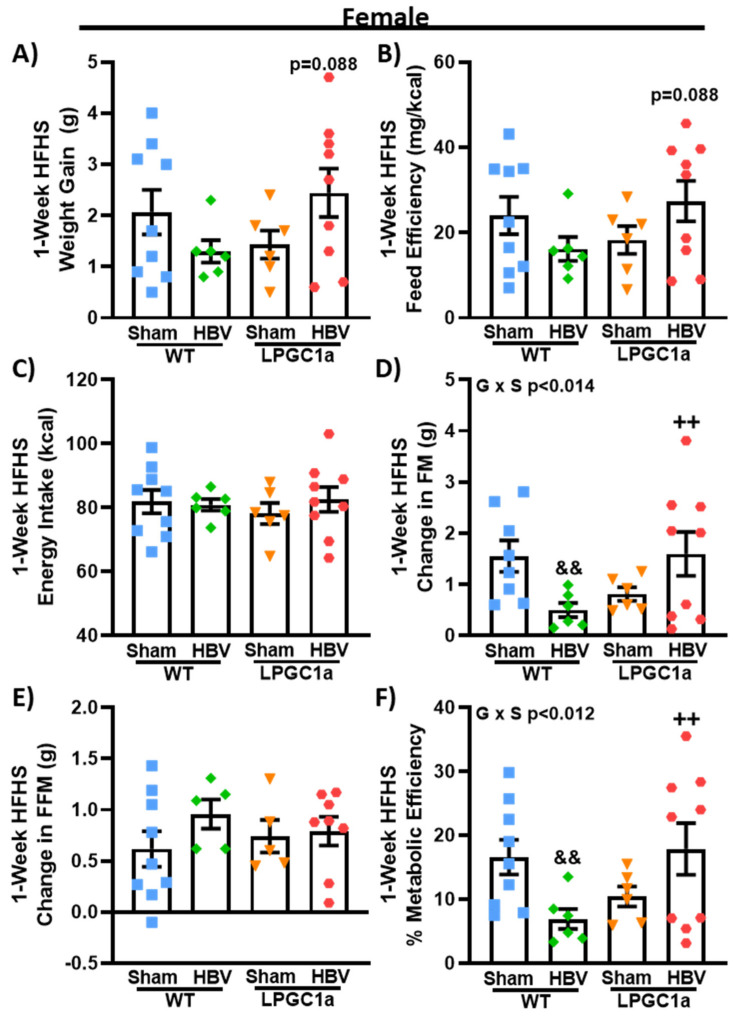
Common hepatic branch vagotomy reduces HFHS-induced fat mass gain in female WT, but not LPGC1a, mice. As with males, female WT and LPGC1a mice underwent sham or common hepatic branch vagotomy surgery. (**A**) Weight gain, (**B**) feed efficiency, (**C**) energy intake, change in (**D**) fat- and (**E**) fat-free mass, and (**F**) metabolic efficiency were determined following one week of HFHS feeding. Individual data points and the group means ± SEM are displayed (n = 6–9). Genotype x Surgery *p* < 0.05 interaction of genotype and surgery, && *p* < 0.05 sham vs. HBV within genotype, ++ *p* < 0.05 WT vs. LPGC1a within surgery.

To determine if there are sexually dimorphic contributions of the HBV on LPGC1a-mediated weight gain changes during HFHS feeding, we performed HBV or sham operations in female WT and LPGC1a mice. As with males, there was no difference between HBV and sham mice at the baseline or during the LFD. While not significant, HBV produced a divergent HFHS-induced weight gain in female WT and LPGC1a mice (Figure 8A, *p* = 0.088, WT HBV vs. LPGC1a HBV). The observed weight gain was the main effector of the trend toward differences in feed efficiency (Figure 8B, *p* = 0.081, WT HBV vs. LPGC1a HBV), as no difference in the one-week HFHS energy intake was observed by genotype or surgery (Figure 8C). There was a 65% less HFHS-induced fat mass gain in female HBV WT mice compared to sham WT mice (*p* < 0.05), with HBV LPGC1a mice gaining 3.2-fold more fat mass than HBV WT mice (*p* < 0.05). Interestingly, unlike the male mice (Figure 7E), no difference was observed in the HFHS-induced fat-free mass gain by genotype or surgery in female mice (Figure 8E). These data suggest that HBV signaling is important in the partitioning of macronutrients to adipose tissue in female mice.

## 4. Discussion

Herein, we present the novel finding that the reduced hepatic PGC1a expression resulted in increased short-term HFHS-induced weight gain in male, but not female, mice. This increased diet-induced weight gain in male LPGC1a mice was associated with altered feeding patterns and reduced activity EE. Further, the disruption of hepatic vagal innervation via common hepatic branch vagotomy increased short-term diet-induced weight gain in male WT mice with no observed difference in male LPGC1a mice.

The role of the liver in systemic energy homeostasis has largely been ascribed to the maintenance of blood glucose levels, triglyceride/apolipoprotein metabolism, ketogenesis, and insulin clearance. Additionally, it has previously been observed that the energy state of the liver and vagal innervation are also critical for regulating insulin secretion and acute food intake regulation (32, 45). Further, an inverse relationship between the liver-specific modulation of fatty acid oxidation and mitochondrial energy metabolism [33,34,35,36] or associated secondary changes in liver FAO and mitochondria gene expression has been observed with chronic diet-induced weight gain [37,38]. However, others have observed an increased food intake in mice with increased liver FAO following the overexpression of CPT-1 [39]. Our previous findings suggested that the differences in acute HFHS-induced weight gain, body composition changes, and the regulation of food/energy intake are inversely associated with liver tissue PGC1a expression, fatty acid oxidation, and mitochondrial respiratory capacity [40,41,42]. To specifically assess the role of liver mitochondrial fatty acid oxidation and respiratory capacity on short-term HFHS-induced changes in energy homeostasis, we utilized a liver-specific, *pgc1a* heterozygous mouse model that has previously been observed to have excess lipid accumulation, a reduced expression of fatty acid oxidation, and mitochondrial genes [18].

The mechanistic control of energy homeostasis during each short-term exposure to energy dense foods represents a complex interaction of central and peripheral processes. It has been postulated that liver energy metabolism is involved in this regulation [43]. Herein, we showed that liver-specific decreases in fatty acid oxidation and mitochondrial respiratory capacity are associated with increased short-term diet-induced weight gain in male mice. As expected, the 3 day HFHS diet resulted in a more positive energy balance in all groups, which was driven primarily by the increased energy intake across the 3 day feeding compared to the LFD. However, the increased diet-induced weight gain of the male HFHS-fed LPGC1a mice was supported by a further 35% more positive energy balance compared to WT males. This more positive energy balance resulted from both a slightly higher energy intake paired with an impaired ability to increase energy expenditure on the HFHS diet. Female LPGC1a mice were not observed to have an altered short-term diet-induced weight gain or energy balance compared to WT mice. Thus, it appears that liver energy metabolism is a peripheral mediator of adaptive homeostatic responses to energy dense diet exposure in male mice.

Previously, we and others have observed reduced food/energy intake in models with increased systemic and liver fatty acid oxidation [40,44,45]. Earlier findings supported a role for the liver in food intake regulation as an association of the chemical inhibition of hepatic fatty acid oxidation and/or reduction in cellular ATP levels with an increased acute food intake [13,14,16,17,46]. However, this work was confounded by the potential off-site action of the compounds through intraperitoneal delivery. Herein, our observations of an increased HFHS intake and the differences in HFHS feeding patterns in a male mouse model of liver-specific alterations in mitochondrial respiratory capacity and fatty acid oxidation suggest the liver is involved in food intake regulation. Interestingly, the reduced number of feeding bouts and the increased time between feeding bouts suggests that the male LPGC1a mice had an improved satiety regulation during the HFHS feeding compared to the WT mice, preventing the beginning of the next feeding bout. While the length of the feeding events for both male LPGC1a and WT mice was similar, the increased consumption within each feeding bout suggests a reduced sensitivity to satiation signals and impaired meal termination. However, it cannot be discerned whether peripheral satiation hormone signaling, gastric mechanoreceptor signaling, or both may be involved in the altered food intake regulation and increased diet-induced weight gain of male LPGC1a mice (reviewed in [47]). All told, these data implicate liver energy metabolism in the regulation of acute food intake patterns during exposure to a HFHS diet, but further experiments are necessary to ascertain why these differences are only observed in males, during HFHS feeding, and what peripheral satiation signaling mechanisms are involved.

Peripheral tissue EE represents the bulk of the total EE and has a large capacity to change through alterations in activity EE and to adapt to environmental stressors such as cold and diet. The diet-induced adaptive response in EE to energy dense feeding occurs as an increased resting EE through a centrally mediated increase in peripheral non-shivering thermogenesis [48]. Importantly, both rodent models [49,50] and humans [51,52] predisposed to diet-induced weight gain and obesity have a reduced diet-induced adaptation of EE. Throughout the study, female LPGC1a mice had a comparable change in total and resting EE compared to WT mice during HFHS feeding. However, male LPGC1a mice did not have a greater total EE during the HFHS feeding compared to the LFD. This appears to be entirely due to a substantial reduction in activity EE and cage activity during the 3 day diet intervention. However, it is unclear how acute HFHS feeding reduces activity and activity EE in male LPGC1a mice. While other liver-specific genetic models have demonstrated associated increases in the total EE and reduced diet-induced weight gain [33,53], these data represent the first observations of an impaired adaptation of total EE due to reduced activity and activity EE during HFHS feeding.

The role of peripheral afferent signaling via the vagus nerve in food intake regulation (reviewed in [47]) and diet-induced obesity (reviewed in [54]) has been well described. Recent data in rats [55,56], as well as in human clinical trials [57,58] implicate vagal stimulation as a treatment for obesity as it reduces body weight, fat mass, and food intake. Food intake regulation is altered through vagal activity by numerous peripheral stimuli, while controversial, hepatic fatty acid oxidation and energy status have been purported to impact food intake regulation via afferent vagal signals [15,16]. Hepatic vagal afferent signaling has been proposed to regulate the efferent vagal control of pancreatic insulin secretion [31,32]. Recently, increased hepatocyte lipid accumulation has been associated with a decreased ATP and membrane depolarization, which was observed to decrease the hepatic vagal afferent signal [59]. This decrease in the vagal afferent signal appears to be due to the increased hepatocyte secretion of GABA due to decreased Na+/K+-ATPase activity. Alternatively, chronic hepatocyte membrane hyperpolarization resulted in reduced chronic high-fat diet-induced weight gain. To assess if the observed increase in acute diet-induced weight gain of male LPGC1a mice was due to the vagal signaling of reduced hepatic fatty acid oxidation and mitochondrial respiratory capacity, we performed HBV in WT and LPGC1a mice. While the vagotomy had no impact on short-term HFHS-induced weight gain in male LPGC1a mice, it did produce increased weight gain in WT littermates. Previously, HBV produced greater weight gain in rats fed a HFD for 3 days compared with sham, with no difference in energy intake per body weight observed [60]. More recently, vagal deafferentation resulted in no difference in ad lib low-fat diet food intake and weight gain on chow, but increased food intake and weight gain on a HFD [61]. Further, this increase in diet-induced weight gain in WT male mice could be related to the loss of the gastroduodenal vagal afferent signals communicating small intestine dietary lipid sensing [62], which adapts lipid metabolism during high-fat diet exposure, similar to those used in our experiments [63]. While the male WT findings support the role of peripheral vagal signaling in diet-induced weight gain, the lack of impact of HBV on male LPGC1a weight gain does not exclude a potential role of hepatic vagal afferents in response to HFHS diets in male LPGC1a mice. Future studies on the role of peripheral vagal afferent signaling on acute diet-induced weight gain that utilize a more nuanced methodology to manipulate vagal signaling are needed. Unlike WT HBV males, no increase in short-term diet-induced weight gain was observed in HBV female WT mice. Interestingly, HBV in female LPGC1a mice resulted in greater weight gain compared to WT mice, which occurred without any change in energy intake. This suggests changes in systemic metabolism and nutrient partitioning in female LPGC1a mice following HBV, which is further supported by the observed differences in metabolic efficiency. While it is currently unknown what role vagal signaling and liver mitochondrial energy metabolism play in female mouse diet-induced weight gain, these findings highlight the need for more sex-specific and comparative studies assessing the regulation of energy homeostasis.

The limitations of the study include the use of HBV, which eliminates the predominantly peripheral afferent and to a lesser extent, some efferent vagal function for all neurons of the hepatic branch proper and the gastroduodenal branch. The above findings in the HBV studies highlight the need for more precise deafferentation techniques to specifically assess the role of the hepatic branch vagal afferents in energy homeostasis. This potentially confounds our findings by the loss of afferent gastroduodenal signaling (e.g., CCK, gastric mechanoreceptors) in food intake regulation and the loss of efferent regulation of hepatic glucose output. Moreover, the 3 day HFHS studies represent a relatively short exposure time to the diet in mice and may have led to increased variability across numerous outcome measures. Future studies should utilize the one-week HFHS feeding paradigm as utilized in the HBV experiments. Moreover, the lack of FAO and mitochondrial respiration data in female mice makes it impossible to fully assess any role of these outcomes in the female diet-induced weight gain phenotype. In addition, the study was under-powered to allow for the direct comparison of males and females. Additionally, no determination of the female hepatocyte mitochondrial energy metabolism was performed. We observed similar hepatocyte mitochondrial respiratory capacities in female WT and LPGC1a mice [64], which could explain the lack of differences in acute diet-induced weight gain. The differences in sex-based responses by genotype and HBV to the HFHS exposure highlights the need for future studies powered for direct sex comparisons. Further, the potential role of a vagal afferent signal communicating liver FAO and mitochondrial energy metabolism to the CNS may be reduced by the limited evidence of vagal afferent innervation of the liver parenchyma [65]. Alternatively, the systemic metabolic effects of the reduced liver FAO and mitochondrial energy metabolism may be due to altered hepatokine secretion (reviewed in [66]). Finally, the discussion of any vagal communication of decreased liver FAO and mitochondrial energy metabolism is further complicated by the possibility of the non-hepatocyte activation of the albumin promoter driven *cre* recombinase, particularly in the newborn rodent kidney and pancreas [67], and the rodent small intestine [68]. Whether the LPGC1a mouse has a reduced PGC1a expression in the kidney or small intestine is unknown. The potential of a decreased FAO in the small intestine is particularly compelling considering the evidence that enterocytes sense dietary fat via FAO and may alter food intake through vagal afferent signaling (reviewed in [62]). However, the stimulation of the vagal afferent signal was observed following the portal infusion of the FFA and lipid emulsion [69], further suggesting that liver lipid metabolism may impact energy homeostasis through vagal signals. Future studies to continue these investigations should utilize the thyroxine binding globulin promoter driven *cre* recombinase delivered via the AAV serotype 8 to heterozygous floxed *pgc1a* mice to more specifically target the reduction of the hepatocyte FAO and mitochondrial energy metabolism [70]. Additional studies should utilize heterozygous flox *cpt1a* and *tfam* mice to specifically target hepatocyte FAO and mitochondrial energy metabolism, respectively.

## 5. Conclusions

In conclusion, we observed that male, but not female mice with reduced hepatocyte mitochondrial fatty acid oxidation and respiratory capacity due to the heterozygosity of PGC1a are more susceptible to acute diet-induced weight gain during HFHS exposure. This greater weight gain occurred with an associated greater energy balance due to the subtle increase in food intake and the lack of a diet-induced adaptation of the total EE. This lack of EE adaptation in male LPGC1a mice resulted from a large drop in activity and activity EE during the HFHS exposure. HBV did not prevent or accentuate this HFHS weight gain in male LPGC1a mice, but it did result in greater short-term diet-induced weight gain in WT males and LPGC1a females. Overall, these data demonstrate a sex-specific impact of reduced liver energy metabolism on diet-induced weight gain and highlight the need for more specific vagal deafferentation studies and a direct sex comparison of the interaction between liver energy metabolism and vagal afferent signaling in energy homoeostasis.

## Figures and Tables

**Table 1:** Primers.

Genotyping		Forward Primer	Reverse Primer
**Tail**	**LoxP sites**	TCC AGT AGG CAG AGA TTT ATG AC	TGT CTG GTT TGA CAA TCT GCT AGG TC
	**Alb-cre**	TTA GAG GGG AAC AGC TCC AGA TGG	GTG AAA CAG CAT TGC TGT CAC TT
**Liver**	**WT PGC1a**	CCA GTT TCT TCA TTG GTG TG	ACC TGT CTT TGC CTA TGA TTC
	**Mutant PGC1a**	TCC AGT AGG CAG AGA TTT ATG AC	CCA ACT GTC TAT AAT TCC AGT TC
**RT-PCR**			
PGC1a (exon 3–5)		AGC CGT GAC CAC TGA CAA CGA G	GCT GCA TGG TTC TGA GTG CTA AG

**Table 2:** Initial anthropometrics.

	WT		LPGC1a	
3-Day Weight Gain Experiments	LFD	HFHS	LFD	HFHS
Body Weight				
Male	30.1 ± 0.8	28.9 ± 1.1	31.8 ± 1.4	31.2 ± 1.0
Female	22.7 ± 1.0	23.9 ± 1.0	23.3 ± 0.9	22.7 ± 0.8
**HBV Experiments 7-Days HFHS**	**Sham**	**HBV**	**Sham**	**HBV**
Male				
Body Weight	25.5 ± 0.6	24.9 ± 0.3	27.0 ± 0.6	24.5 ± 0.4 ^&&^
Fat Mass	2.54 ± 0.33	2.40 ± 0.24	2.88 ± 0.54	2.19 ± 0.10
Fat-Free Mass	22.96 ± 0.69	22.44 ± 0.33	22.91 ± 0.44	22.25 ± 0.37
Female				
Body Weight	20.2 ± 0.3	19.7 ± 0.1	20.1 ± 0.1	20.3 ± 0.6
Fat Mass	1.52 ± 0.09	1.39 ± 0.08	1.32 ± 0.12	1.79 ± 0.24
Fat-Free Mass	18.70 ± 0.27	18.35 ± 0.11	18.80 ± 0.24	18.48 ± 0.43

&& *p* < 0.05 Sham vs. HBV within genotyped. Sham—sham surgery, HBV—hepatic common branch vagotomy.

## Data Availability

Some or all datasets generated during and/or analyzed during the current study are not publicly available but are available from the corresponding author on reasonable request.
